# Relationships Between Disc Degeneration and Autophagy Expression in Human Nucleus Pulposus

**DOI:** 10.1111/os.12573

**Published:** 2019-12-04

**Authors:** Meiling Quan, Myoung‐Wha Hong, Myung‐Sup Ko, Young‐Yul Kim

**Affiliations:** ^1^ Department of Orthopedics Daejeon St. Mary's Hospital, College of Medicine Daejeon Korea; ^2^ Department of Orthopedics College of Medicine, The Catholic University of Korea Seoul Korea

**Keywords:** Autophagy, Degenerative grade, Disc degeneration, LAMP, LC3

## Abstract

**Objectives:**

To elaborate on the relationship between degeneration grade and autophagy expression in human nucleus pulposus obtained from surgical procedures.

**Methods:**

For the 16 patients included in the present study, we determined the Pfirrmann classifications of degenerative lesions by MRI. Western blot analysis, LC3, LAMP2, and p62 protein expressions were quantified in different degeneration grades of disc nucleus pulposus. Double immunofluorescence staining was used to show co‐localization of LC3 and LAMP2, and immunohistochemistry to show LC3 and p62 in the nucleus pulposus.

**Results:**

In the western blot analysis, LC3‐II was highly expressed in grade III and decreased progressively from grade IV to V. In addition, LC3‐II expression in grade III was significantly higher than in grade II, IV, and V (*P* < 0.05). LAMP2 expression in grade V was significantly higher than that in grade II, III, and IV (*P* < 0.05). P62 increased with decreasing autophagy expression up through grade V. In the double staining, the LC3 level was highly expressed in grade III and decreased progressively from grades IV to V, as in the western blot analysis. LAMP2 rose with increasing disc degeneration grade through grade V. In the quantitative analysis of colocalization, grade III is significantly higher than grade II and V (*P* < 0.05). Immunohistochemical staining showed that LC3 was highly expressed in grade III but weakly expressed in other grades, with few positive areas around the nucleus pulposus. However, p62 increased progressively with increasing disc degeneration grade.

**Conclusion:**

Pfirrmann grade III disc degeneration showed that autophagosomes were formed, which led to the reversible decomposition of degenerative substances. Thus, by analyzing the effect of autophagy on degenerative discs, we showed the possibility of reversing degenerative changes, but only in grades III and lower.

## Introduction

Intervertebral disc degeneration (IDD) is the pathological basis of various degenerative diseases of the spine^1^. IDD is a major contributor to low back and neck pain. It is a pathological and physiological process that can be chronic or progressive^1^, ^2^. IDD, from excessive external stress, hereditary diseases, obesity, and aging, leads to increased intervertebral disc (IVD) cell death and loss of IVD cellular functions^3^. Furthermore, changes and degradation of extracellular matrix (ECM) contents, such as proteoglycan protein, are decreased by matrix metalloproteinases^4^. IVD are almost completely avascular and aneural, and consists of three components: the central gelatinous nucleus pulposus, the annulus fibrosus, and upper and lower cartilaginous endplates^5^, ^6^. Because of this structural composition, IVD has biomechanical properties to maintain spinal flexibility and mechanical stability, which tends to degraded over time^7^. Such degradation presents great challenges for researchers and the current treatment of disc degeneration focuses on the homeostasis of the ECM and loss of cell number and function^3^.

Previously, disc degeneration was believed to occur with disc cell apoptosis, which causes programmed cell death; however, recent studies have focused on autophagy function, such as IDD,^8^ diabetes,^9^ neurodegeneration,^10^ and aging^11^.

Apoptosis, known as type I programmed cell death, is characterized by caspase activation, cell shrinkage, nuclear and cytoplasmic condensation, DNA fragmentation, and formation of apoptosomes^12^. Apoptosis has been shown to participate in IVD degeneration, and several studies have found that nucleus pulposus cells undergo apoptosis in degenerative discs through complicated mechanisms^13^. Wang et al.^14^ reported that nucleus pulposus cells treated with IL‐1β appeared to have increased production of nitric oxide and decreased levels of proteoglycan, which led to apoptosis. Yang et al.^15^ reported increased apoptosis in nucleus pulposus cells treated with pro‐oxidant H_2_O_2_ as well as decreased mRNA levels of matrix proteins aggrecan and type II collagen.

**Figure 1:**
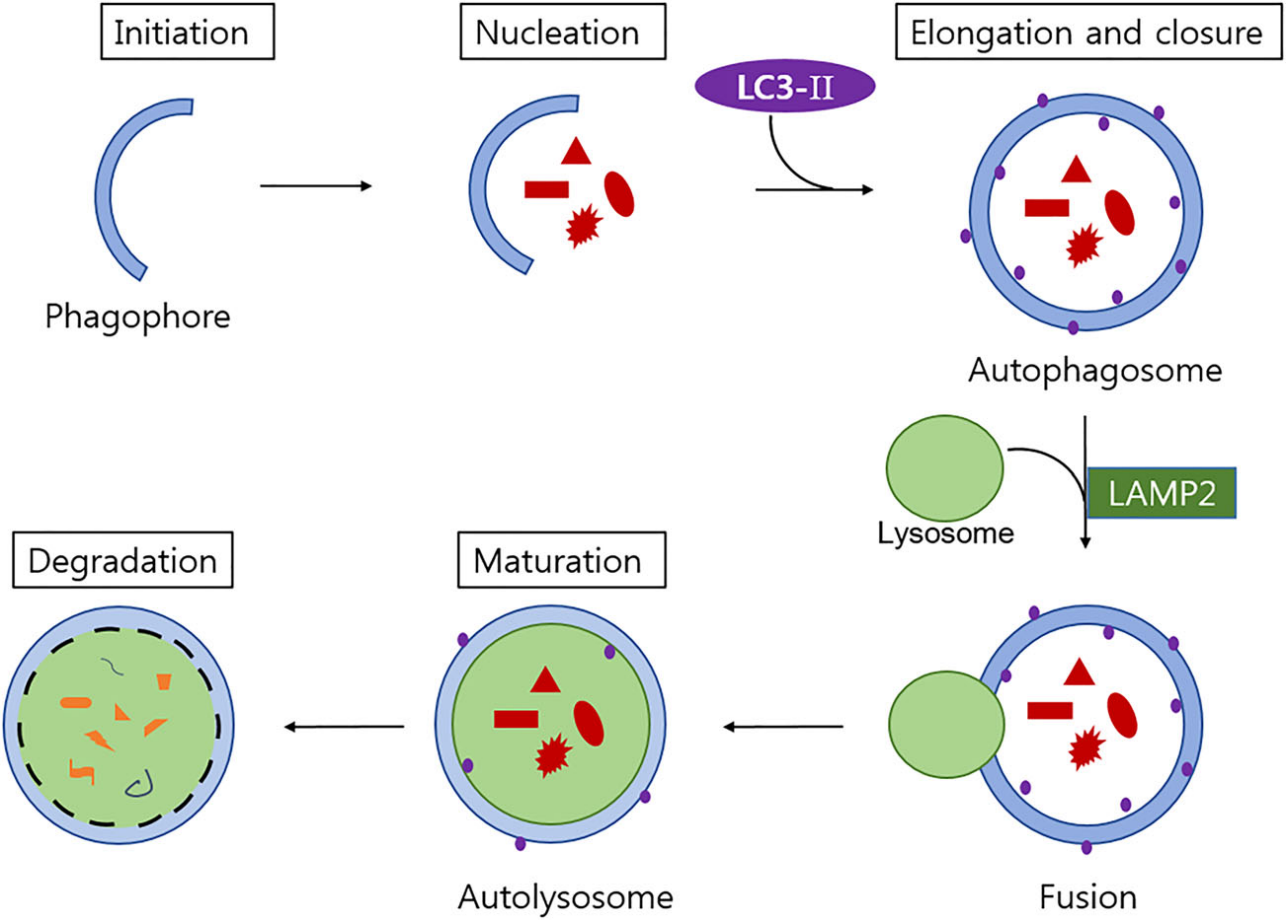
Schematic diagram of autophagy. Autophagy contains initiation, nucleation, elongation and closure (formation of autophagosome), maturation (formation of autolysosome), and degradation of vesicle contents

Autophagy is an evolutionally conserved lysosomal activity that degrades and turns over long‐lived proteins and damaged cytoplasmic organelles^12^. Since the discovery of autophagy, it has been thought to act as a pro‐survival response to several stresses by providing recycled metabolic substrates to maintain energy homeostasis^12^, ^16^. Although primarily cytoprotective, autophagy also contributes to cell death, thereby providing an alternative caspase‐independent cell death mechanism called type II programmed cell death^7^. Autophagy is different from apoptosis because of cell recycling rescue processes. The procedure of autophagy is shown in Fig. 1. It begins with the autophagosome membrane that completes membrane elongation along with participating microtubule‐associated protein 1A/1B light chain 3 (LC3), which is crucial.^17^ The newly synthesized LC3 is produced by Atg 4 and is mainly present in the cytoplasmic form LC3‐I. When autophagy is induced, LC3‐I conjugates with phosphatidylethanolamine and converts to LC3‐II, which is recruited to the autophagosome membrane^18^. The mature autophagosome fuses with a lysosome to form an autolysosome, and the isolated cytoplasmic material is degraded with the help of lysosome‐associated membrane protein (LAMP)^19^. Autophagy substrate p62 is also degraded by autophagy, and autophagy deficiency may lead to p62 accumulation^12^.

Park et al.^20^ reported that high glucose‐induced oxidative stress promotes autophagy through mitochondrial damage in rat notochordal cells. Shen et al.^4^, Ye et al.^11^, and Ye et al.^21^ have also investigated autophagy in a rat model. Studies on autophagy are actively under way in animal models, whereas research on human subjects in this area is in the early stages, and there have only been a few qualitative studies thus far. In addition, the relationship between autophagy and degenerative diseases is controversial, and the precise mechanisms for these diseases are unknown^7^.

The purpose of this study is: (i) to identify and classify the degenerative changes of human IVD using the MRI classification system; (ii) to observe the expression of autophagy markers in different degeneration grades; and (iii) to study the relationship between disc degeneration and autophagy.

## Materials and Methods

### *Sample Preparation of Nucleus Pulposus*

Three orthopaedic surgeons decided on the Pfirrmann classification and chose coincidental agreement of classification at fusion, and we gathered 35 cases of nucleus pulposus samples but removed 19 cases because there were ambiguous classifications which did not match each other. A total of 16 samples were finally gathered and evaluated (male: 10 [62.5%]; female: 6 [37.5%]; age: 61.44 ± 11.14). Intervertebral nucleus pulposus tissue was taken from patients with degenerative disc disease who underwent surgical disc procedures. The specimens were immediately transported to the laboratory after surgery and placed in separate cryotubes and 5‐mL tubes containing a 10% formalin solution. These tissues in cryotubes were stored at −80°C.

All experiments were performed with the approval and guidance of the Institutional Review Board (DC18SESI0059). The clinical and demographic features of the population used in the molecular and morphological studies are presented in Table 1.

**Table 1:** Summary of demographic features for the study population

Subject number	Age (year)/sex	Vertebral level	Pfirrmann grade	Surgery information
1	F/42	L_4–5_ disc	II	Trauma/posterior instrumentation
2	M/51	L_4–5_ disc	II	Trauma/anterior corpectomy
3	F/52	L_5_‐S_1_ disc	II	Spondylolysis/Posterior fusion
4	F/50	L_4–5_ disc	II	Spondylolisthesis/PLIF
5	F/48	L_3–4_ disc	III	Spinal stenosis/laminectomy and discectomy
6	M/54	L_3–4_ disc	III	Spondylolisthesis/PLIF
7	M/57	L_4–5_ disc	III	Trauma/posterior instrumentation and decompression
8	M/76	L_3–4_ disc	III	HNP/ACDF
9	F/61	L_4–5_ disc	IV	Spinal stenosis/PLIF
10	M/72	L_3–5_ disc	IV	Spinal stenosis/PLIF
11	F/67	L_5_‐S_1_ disc	IV	HNP/discectomy
12	F/69	L_3–4_ disc	IV	Spinal stenosis/PLIF
13	F/75	L_4–5_ disc	V	Spinal stenosis/PLIF
14	F/77	L_5_‐S_1_ disc	V	Spinal stenosis/laminectomy and discectomy
15	F/63	L_4–5_ disc	V	HNP/discectomy
16	M/69	L_4–5_ disc	V	Spondylolisthesis/ PLIF

ACDF, anterior discectomy and fusion; C, cervical; F, female; L, lumbar; M, male; PLF, posterolateral fusion; PLIF, Posterior lumbar interbody fusion; S, sacral.

### *Western Blot Analysis*

Tissues were homogenized using a TissueLyser (TissueLyser II, QIAGEN, Germany) at 30 times/min for 3 min in RIPA buffer (CBR0002 [LPS Solution, Korea]), with cOmplete tablets and an EDTA‐free protease inhibitor cocktail (0469332001 [Sigma‐Aldrich, Germany]). After tissue lysate tubes were placed on ice and allowed to cool, soluble proteins were collected by performing 16128 g cold centrifugation for 15 min. Protein concentrations were measured using a Bradford protein assay (Bio‐Rad Laboratories, USA).

Twenty‐microgram amounts of protein were mixed with the electrophoresis sample buffer and boiled at 95°C for 5 min. Proteins were separated by 4%–15% sulfate–polyacrylamide gel electrophoresis (#456‐1083 [Bio‐Rad Laboratories, USA]). The separated proteins were transferred to a nitrocellulose membrane (P/N 66485 [PALL Life Sciences, Mexico]). The membranes were incubated in blocking solution (ProNA blocking solution [TransLab, Korea]) for 60 min, and then incubated with primary antibody rabbit monoclonal anti‐LC3 (1:500, #3868 [Cell Signaling Technology, USA]), mouse monoclonal anti‐LAMP‐2 (1:1,000, ab25631 [Abcam, UK]), mouse monoclonal anti‐p62 (1:1,000, ab56416 [Abcam, UK]), and rabbit monoclonal anti‐β‐actin (1:3,000, #5125 [Cell Signaling Technology, USA]) overnight at 4°C. The next day, the membranes were washed three times in TBST and incubated with the second antibodies of horseradish peroxidase‐linked anti‐rabbit immunoglobulin (1:2,000, #7074 [Cell Signaling Technology, USA]) and horseradish peroxidase‐linked anti‐mouse immunoglobulin G (1:2,000, #7076 [Cell Signaling Technology, USA]) for 2 h at room temperature. After washing in TBST, the membranes were detected using ECL (iNtRON Biotechnology, Korea).

### *Double Staining*

Paraffin‐embedded sections were deparaffinized in xylene and hydrated in graded ethanol. The slides were washed with phosphate‐buffered saline (PBS) and blocked in blocking buffer (1× PBS/5% bovine serum albumin [BSA]/0.3% triton X‐100) for 60 min. The primary antibody LC3 and LAMP2 were diluted to the recommended concentration (1:200) in 1× PBS containing 1% BSA and 0.3% triton X‐100, and incubated overnight at 4°C. Then, sections were incubated with secondary antibody goat anti‐rabbit IgG H&L (ab150077; Abcam, UK) and goat anti‐mouse IgG H&L (ab150116; Abcam, UK) at room temperature for 2 h in the dark. A mounting medium was used to mount cover slips on the sections to measure fluorescence using 4′, 6‐diamidino‐2‐phenylindole. A super‐resolution confocal laser scanning microscope (LSM880 with Airyscan, Zeiss, Germany) was used to examine the sections. The quantitative colocalization analyses were performed with ImageJ software (v1.46 software, NIH Image, USA).

### *Immunohistochemistry*

The sections were deparaffinized in xylene, hydrated in graded ethanol, and washed for 5 min in tap water. The sections were rinsed in distilled water and treated for antigen retrieval using a citrate buffer. Then, immunohistochemical analysis using a Vectastain ABC Kit (Vector Laboratories, USA) was performed according to the manufacturer's instructions, and the sections were incubated with normal blocking serum and the primary antibodies LC3 (#3868; Cell Signaling Technology, USA) and p62 (ab56416; Abcam, UK) overnight. The next day, the sections were incubated for 1 h with a biotinylated secondary antibody. The sections were examined using the ImmPACT NovaRED peroxidase substrate (Sk‐4805; Vector Laboratories, USA).

### *Statistical Analysis*

All experiments were performed independently three times, and all results are presented as means ± standard deviations. The western blotting and double staining data were analyzed using Kruskal–Wallis variance analysis and the Mann–Whitney *U*‐test in SPSS 19.0 software (Chicago, IL, USA). *P‐*values less than 0.05 were considered statistically significant.

## Results

### *Expression of LC3 in Different Grades of Human*

To observe the relationship between the occurrence of autophagy and the degenerated disc grade, we analyzed the expression levels of autophagy marker LC3.

**Figure 2:**
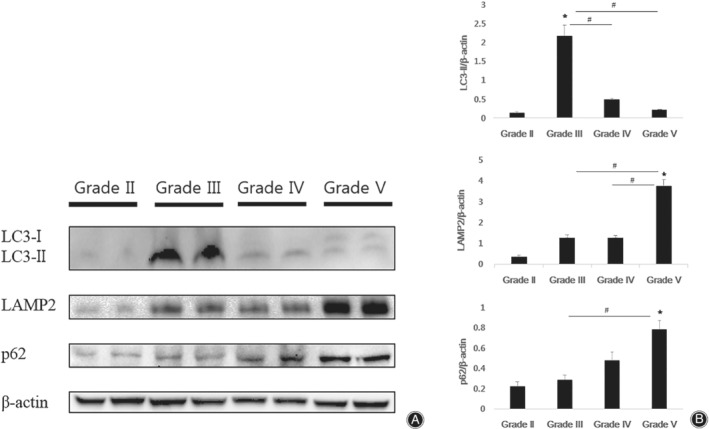
Western blot analysis (A) and density measurement (B) of the autophagic flux markers LC3, LAMP2, and p62. LC3‐II was highly expressed in Pfirrmann grade III and decreased progressively from Pfirrmann grade IV to V. LAMP2 was least expressed in Pfirrmann grade ǁ and increased gradually with increasing Pfirrmann grade. P62 was present in Pfirrmann grades II–V and increased with increasing disc degeneration grade (*P < 0.05 *vs* Pfirrmann grade II, and # *P* < 0.05 was used to compare different groups)

LC3 is a protein required in the formation of autophagosomes and it is converted to LC3‐II by lipidation of LC3‐I. In our results, LC3‐I was found in Pfirrmann grades IV and V, and Pfirrmann grade V was higher than Pfirrmann grade IV. LC3‐II expression was the lowest in Pfirrmann grade II, greatest in Pfirrmann grade III, and decreased gradually in Pfirrmann grades IV and V (Fig. 2A). Quantitative analysis showed that the expression levels of LC3‐II in Pfirrmann grade III, IV, and V were 17.17‐fold, 3.85‐fold, and 1.62‐fold of Pfirrmann grade II, respectively. In addition, LC3‐II expression in Pfirrmann grade II was significantly different from that in Pfirrmann grade III (*P* < 0.05). The expression level of LC3‐II in Pfirrmann grade III (4.46‐fold of grade IV, 10.63‐fold of grade V) was also significantly different from those in Pfirrmann grades IV and V (*P* < 0.05, Fig. 2B).

### *Expression of LAMP2 and p62 in Different Grades of Human Degenerated Disc Nucleus Pulposus*

To further observe the occurrence of autophagy in different grades of degenerated discs, we analyzed the expression levels of LAMP2 and p62. LAMP2 degrades autophagosomes and is essential for autophagy generation. LAMP2 was present in all Pfirrmann grades but was least expressed in Pfirrmann grade II and increased gradually with increasing Pfirrmann grade (Fig. 2A). The expression levels of LAMP2 in Pfirrmann grade V was 10.54‐fold of grade II, 3.00‐fold of grade III, and 2.95‐fold of grade IV (*P* < 0.05, Fig. 2B).

P62 is an important marker in the development of autophagy and has been found in Pfirrmann grades II, III, IV, and V. The expression level of p62 increased with increasing disc degeneration grade and was the highest in Pfirrmann grade V (Fig. 2A). The expression levels of p62 in Pfirrmann grades V was 3.51‐fold of grade II (*P*< 0.05), 2.70‐fold of grade III (*P*< 0.05), and 1.63‐fold of grade IV (Fig. 2B).

### *LC3 and LAMP2 Co‐localization in Different Grades of Human Degenerated Disc Nucleus Pulposus*

**Figure 3:**
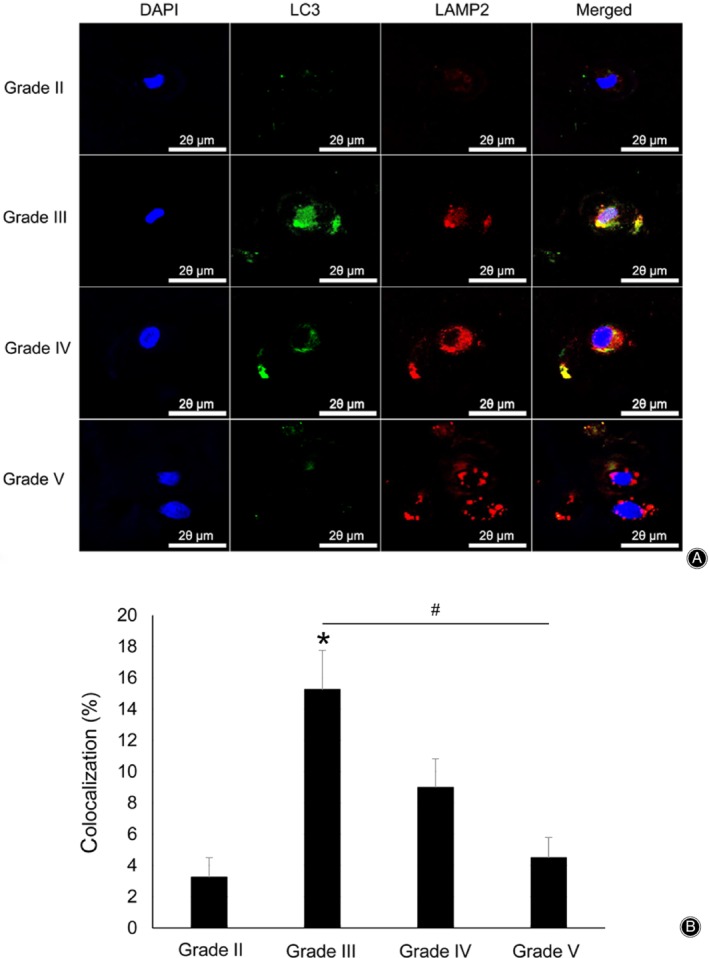
Double immunofluorescence of LC3 (green) and LAMP2 (red) (×1600) (A) and quantitation analysis of colocalization (B). The LC3 level was highly expressed in Pfirrmann grade III and decreased progressively from Pfirrmann grade IV to V. LAMP2 increased with increasing disc degeneration grade. Colocalization of LC3 and LAMP2 was the highest in Pfirrmann grade III and was mainly observed around the nucleus and cell membrane (*P < 0.05 *vs* Pfirrmann grade II, and # *P* < 0.05 was used to compare different groups)

To further analyze the degree of autophagy degradation, co‐localization of LC3 and LAMP2 was confirmed by double immunofluorescence labeling with LC3 and LAMP2 antibodies (Fig. 3).

### *Co‐localization in Pfirrmann Grade II*

In Pfirrmann grade II, a small amount of LC3 was present, shown by a pattern of fluorescent dots around the nucleus of nucleus pulposus cell, and LAMP2 showed a similar pattern, but with fewer fluorescent dots around the nucleus of nucleus pulposus cell (Fig. 3A). Quantitative analysis of co‐localization showed that their co‐localization percentage was 3.25% (Fig. 3B).

### *Co‐localization in Pfirrmann Grade III*

In Pfirrmann grade III, LC3 was significantly increased and distributed mainly around the nucleus and membrane of the nucleus pulposus cell. LAMP2 was higher in Pfirrmann grade III than in grade II, and mainly distributed around the cell nucleus and membrane. The distributions of LC3 and LAMP2 were similar, and co‐localization was more frequent in Pfirrmann grade III than in the other Pfirrmann grades, mainly peripherally around the nucleus and membrane of the nucleus pulposus cell (Fig. 3A). Quantitative analysis of co‐localization showed that the co‐localization percentage of Pfirrmann grade III was 15.25%, and it was significantly higher than that of Pfirrmann grades II and V (*P* < 0.05, Fig. 3B).

### *Co‐localization in Pfirrmann Grade IV*

In Pfirrmann grade IV, the LC3 level was significantly reduced relative to that in grade III and was distributed mainly around the nucleus and cell membrane of the nucleus pulposus. LAMP2 was slightly higher in Pfirrmann grade IV than in grade III, and its location was mainly around the cell nucleus and membrane. Co‐localization of LC3 and LAMP2 was observed around the cell nucleus and membrane, which was less co‐localized (9.00%) than in Pfirrmann grade III (Fig. 3A and B).

### *Co‐localization in Pfirrmann Grade V*

In Pfirrmann grade V, the LC3 level was significantly reduced relative to that in Pfirrmann grade IV, and LAMP2 was significantly increased relative to that in Pfirrmann grade IV. In the quantitation analysis of colocalization, co‐localization was 4.50% (Fig. 3A and B).

### *Localization of LC3 and p62 in Different Grades of Human Degenerated Disc Nucleus Pulposus*

**Figure 4:**
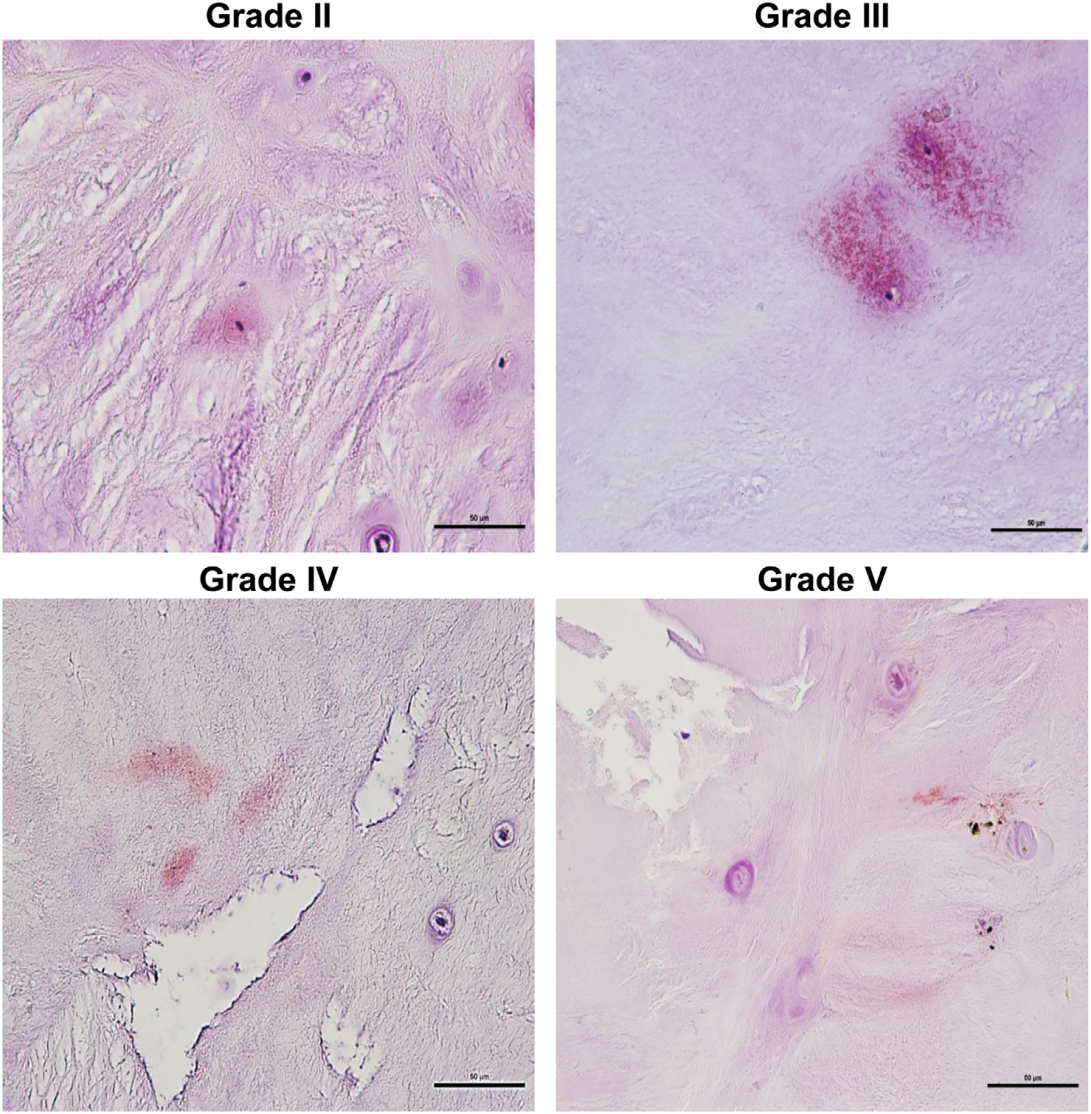
Immunohistochemical analysis showed that LC3 expression (×400) was the highest in Pfirrmann grade III and weak in Pfirrmann grades II, IV, and V, with few positive areas around the nucleus pulposus cells

**Figure 5:**
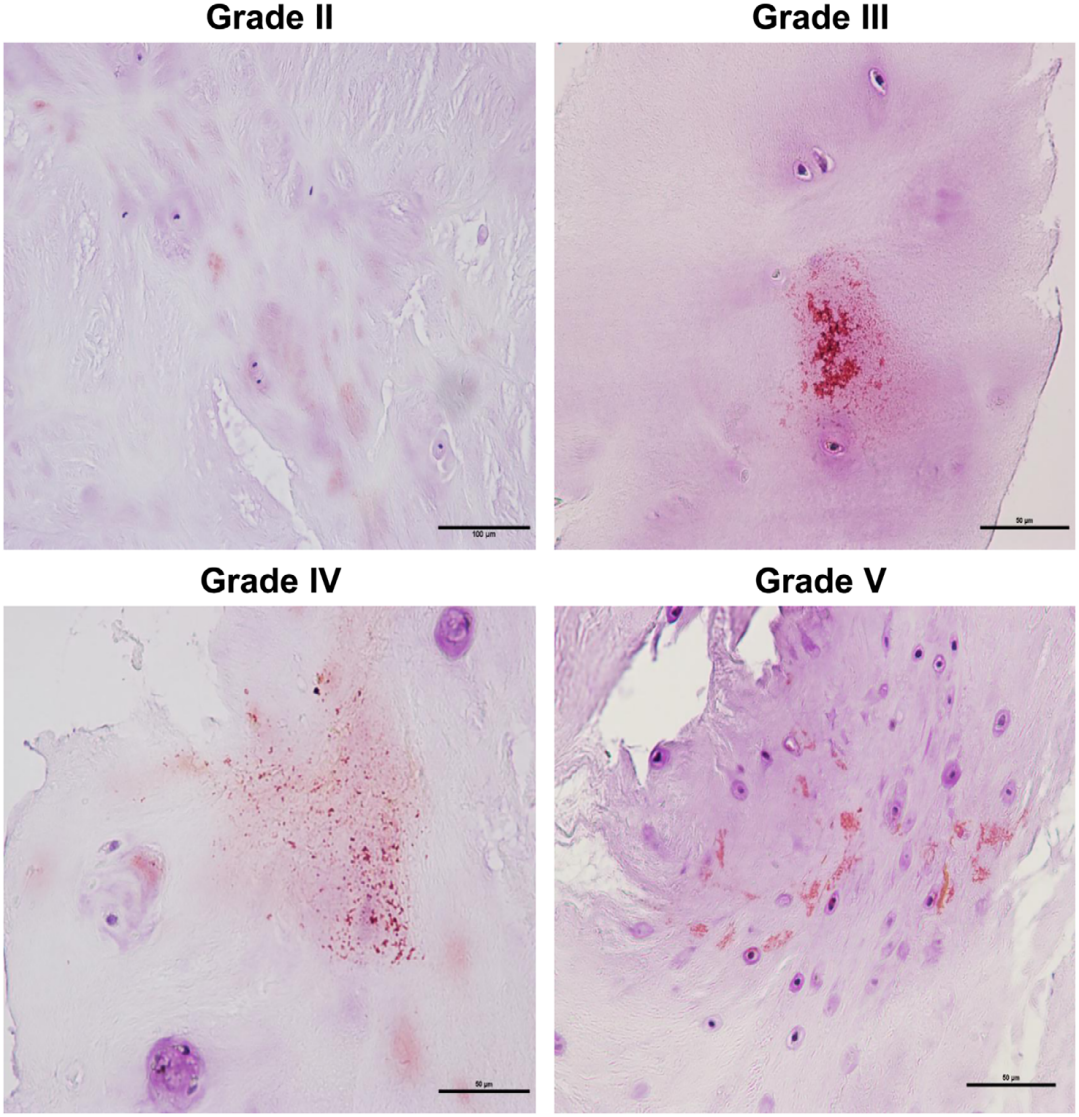
Immunohistochemical analysis of p62 (×400) showed very weak staining in Pfirrmann grade II, with few positive areas around the nucleus pulposus cells, but increased progressively with increasing disc degeneration grade

Localization of p62 and LC3 was observed in disc tissue of various Pfirrmann grades by immunohistochemical staining. The highest expression level of LC3 was observed in Pfirrmann grade III, but grades II, IV, and V showed slight staining, indicating very small amounts. LC3 was localized mainly in the nucleus pulposus cell, cell periphery, and occasionally the matrix (Fig. 4). P62 was stained very weakly in Pfirrmann grade II, and its expression level increased with increasing Pfirrmann grade. This was localized mainly in or around the nucleus pulposus cells (Fig. 5).

## Discussion

As shown in the molecular and morphological results, this study demonstrated the variation of autophagy in different Pfirrmann grades of human degeneration discs for the first time. The disc degeneration stage is referred to as the Pfirrmann grade, determined b/ the MRI classification system (Table 2)^22^.

**Table 2:** Pfirrmann classification of human disc degeneration

Grade	Structure	Distinction between nucleus and annulus	Signal intensity	Height of intervertebral disc
I	Homogeneous, bright white	Clear	Hyperintense, isointense to cerebrospinal fluid	Normal
II	Inhomogeneous, with or without horizontal bands	Clear	Hyperintense, isointense to cerebrospinal fluid	Normal
III	Inhomogeneous, gray	Unclear	Intermediate	Normal to slightly decreased
IV	Inhomogeneous, gray to black	Lost	Intermediate to hypointense	Normal to moderately decreased
V	Inhomogeneous, black	Lost	Hypointense	Collapsed disc space

Autophagy is an intracellular process in which the cytoplasm and cell organs are degraded in an adaptive response to external stress, such as nutrient deprivation, and exists due to avascular and aneural structural features in mature intervertebral discs^23^. Studies have shown that autophagy plays an important protective role in the cell catabolic pathway, leading to lysosomal degradation in aging‐related and degenerative diseases^24^. Wen et al. (2019)^25^ reported that β‐ecdysterone protects disc nucleus pulposus against apoptosis through autophagy activation and ameliorates IDD. Ye et al.^21^ proposed that autophagy was present and associated with increased pathological IDD in rats. Gruber et al.^23^ performed an in vivo gene analysis that showed greater autophagy‐related gene expression in degenerated discs than in healthier discs. However, the abovementioned experiments were not conducted by examining expression according to the degenerative grade. Therefore, in this study, we examined the expression of the important autophagy flux markers LC3, LAMP2, and p62 according to the human degenerative disc grade determined by MRI.

In autophagy, autophagosomes engulf cytosolic proteins and organelles^18^. During the activation of autophagy, LC3‐I (cytosolic form) is conjugated to phosphatidylethanolamine and further cleaved to generate LC3‐II, which results in the association of LC3‐II with autophagosomal membranes. Therefore, LC3 is the only dependable marker of autophagosomes, and the amount of LC3‐II correlates with the degree of autophagosome formation. Zheng et al.^26^ proposed that spermidine protects rat nucleus pulposus cells against apoptosis by autophagy and ameliorates IDD. In this study, LC3‐II expression was increased and the autophagosome number was higher in the spermidine treated group compared to the control group (non‐treated nucleus pulposus). The results suggest that autophagy may affect degenerated discs, but because the comparison is made with a cell‐level animal study, further research is required to reach the human clinical levels. Xu et al.^27^ found that the LC3‐II/LC3‐I ratio was significantly reduced in the cervical spondylosis group relative to that in the cervical vertebral fracture group or the dislocation group with westrn blot analysis. In our western blot analysis, LC3‐II showed the highest expression in Pfirrmann grade III, and the expression levels in the other grades were significantly lower.

Immunohistochemical and immunofluorescence analyses of LC3 showed that it was highly expressed in Pfirrmann grade III, but only weakly expressed in other grades, with few positive areas around the disc cells. These results showed highly increased autophagosome, suggesting that autophagy function remains actively in Pfirrmann grade III. However, as the grade is higher, the formation of autophagosome and activation of autophagy become decreased in a reverse way. They also suggest that degenerated disc tissue could be repaired in Pfirrmann grade III by the formation of autophagosome.

Autophagosomes fuse with lysosomes with the help of LAMP2 to form autolysosomes, and the vesicle contents are degraded and transported to the cytosol^7^. LAMP2 is a lysosomal membrane protein involved in lysosomal stability and autophagy and plays a critical role in the fusion of autophagosomes with lysosomes to form autolysosomes^28^. In humans, mutations in the LAMP2 gene cause Danon's disease, an X‐linked lysosomal storage disorder characterized by the accumulation of vacuolar compartments, leading to cardiomyopathy and myopathy^29^, ^30^. Tanaka et al.^31^ reported that LAMP2‐deficient mice accumulated autophagosomes in many tissues and suggested that LAMP2 is required for degradation or a function in the maturation of the autophagosomes into actively digesting organelles. Therefore, LC3 and LAMP2 were observed to confirm whether the autophagy has occurred smoothly. In our western blot analysis, LAMP2 significantly increased progressively with increasing disc degeneration grade. The immunofluorescence of LAMP2 increased with increasing disc degeneration grade through grade V also, similar to the western blot analysis results. These results indicate that LAMP2 is increased gradually to decompose overformed autophagosomes in Pfirrmann grade III.

p62, also called sequestosome 1 or SQSTM1, is an ubiquitin‐binding scaffold protein that can be degraded by autophagy^32^, ^33^. It recognizes toxic cytoplasmic waste and is removed by a sequestration process known as autophagy^34^. Insufficient autophagy results in accumulation of p62, which is detrimental for the hepatocytes, which induces cellular stress reactions and causes disease^34^. A deficiency in autophagy can damage the ubiquitin–proteasome system due to excessive p62 delaying the transfer of the proteasome substrate to the proteasome, although proteasome catalytic activity is not changed^35^. In our western blot and immunohistochemistry analyses, p62 rose with increasing disc degeneration grade through grade V. p62 accumulation demonstrates that the response of autophagy declines with an increasing degenerative grade. These results indicate that as the Pfirrmann grade increases, the autophagy response gradually weakens and causes autophagy dysfunction in Pfirrmann grade V.

Morell et al.^28^ reported that decreased levels of p62 were accompanied by the enhanced expression of LC3, which suggested the activation of autophagy in neuroendocrine cells. Jiang et al.^24^ reported that LC3‐II was increased in the degenerated disc nucleus pulposus cells of diabetic rats, but p62 protein expression decreased in diabetic rats relative to that in the controls. Similarly, in this study, p62 was less expressed when LC3‐ II was more highly expressed in Pfirrmann grade III, and increased with decreasing LC3‐II expression from Pfirrmann grades IV to V.

Bussi et al.^36^ observed that LC3 showed intracytoplasmic inclusions that co‐localized with the LAMP, which suggested the activation of an autophagic flux. Lee et al.^37^ confirmed that LC3 accumulated in LAMP2‐positive vesicles, which suggested that LC3 degradation after autophagosome–lysosome fusion is impaired. In our double‐stain analysis, co‐localization of LC3 and LAMP2 was the highest in Pfirrmann grade III relative to that in other Pfirrmann grades. This result demonstrates once again that the autophagy response can be reversed in disc degeneration, predominantly in Pfirrmann grade III.

The limitations of this study are that the number of samples was relatively small, a Pfirrmann grade I sample was not obtained, and the number of samples of other Pfirrmann grades was also small.

This study is the first to show that the autophagy response varies with different Pfirrmann grades of human intervertebral discs. In addition, Pfirrmann grade III disc degeneration caused the reversible degradation of degenerative material by forming autophagosomes, but reversible changes caused by autophagy in Pfirrmann grades IV and V were insignificant and did not degrade the substances formed by degenerative discs. Thus, by analyzing the effect of autophagy on degenerative discs, we showed the possibility of reversing degenerative changes only in grades III and lower than III. In summary, the results from this study showed that the therapeutic target of autophagy‐related medicine should be developed to treat the conditions below Pfirrmann grade III.
